# Regulation of peroxisomal trafficking and distribution

**DOI:** 10.1007/s00018-020-03687-5

**Published:** 2020-11-03

**Authors:** Christian Covill-Cooke, Viktoriya S. Toncheva, Josef T. Kittler

**Affiliations:** 1 Present Address: Department of Biochemistry, University of Oxford, Oxford, OX1 3QU UK; 2 Department of Neuroscience, Physiology and Pharmacology, University College London, London, WC1E 6BT UK

**Keywords:** Microtubule, Actin, Disease, Kinesin, Dynein

## Abstract

Peroxisomes are organelles that perform a wide range of essential metabolic processes. To ensure that peroxisomes are optimally positioned in the cell, they must be transported by both long- and short-range trafficking events in response to cellular needs. Here, we review our current understanding of the mechanisms by which the cytoskeleton and organelle contact sites alter peroxisomal distribution. Though the focus of the review is peroxisomal transport in mammalian cells, findings from flies and fungi are used for comparison and to inform the gaps in our understanding. Attention is given to the apparent overlap in regulatory mechanisms for mitochondrial and peroxisomal trafficking, along with the recently discovered role of the mitochondrial Rho-GTPases, Miro, in peroxisomal dynamics. Moreover, we outline and discuss the known pathological and pharmacological conditions that perturb peroxisomal positioning. We conclude by highlighting several gaps in our current knowledge and suggest future directions that require attention.

## Peroxisome homeostasis is important for cellular health

Peroxisomes are single bilayer-membrane bound metabolic organelles that are ubiquitous to eukaryotic life. Their roles vary between species and cell type, though most commonly peroxisomes carry out reactive oxygen species metabolism, β-oxidation of fatty acids and lipid synthesis. The functionality of peroxisomes is strictly dependent on a wide array of homeostatic mechanisms, with the best characterised of these being their biogenesis [1–3]. This process is orchestrated by the *PEX* family of genes with mutations in many members of this family leading to Zellweger spectrum disorder—a group of autosomal recessive diseases that manifest clinically in hepatic and adrenocortical dysfunction and hypomyelination of white matter in the brain [4, 5]. Alongside biogenesis, peroxisomal morphology, turnover and distribution also require dynamic regulation [6]. For example, an individual mature peroxisome can elongate and undergo scission to produce multiple smaller peroxisomes through the process of peroxisomal fission [7]. Mutations in several components of the peroxisomal fission machinery, including Drp1, Mff, GDAP1 and Pex11β, are associated with neurodegenerative diseases [8–12]. Excess or dysfunctional peroxisomes can also be cleared through pexophagy—autophagic engulfment followed by lysosomal degradation—which, in conjunction with fission, controls peroxisomal health and number [13].

The distribution of peroxisomes must be maintained to optimally position peroxisomal functions to where they are required. Like all organelles, the specific localisation of peroxisomes is cell type and condition specific. For example, peroxisomes are homogenously distributed throughout the cytoplasm in mammalian cell lines [14]. Importantly, perturbations in peroxisomal positioning have been shown to be detrimental to cellular health, making cells more susceptible to damage by reactive oxygen species [15]. Many aspects of how peroxisomal trafficking is regulated are poorly understood. Hence, this review aims to give an overview of our current understanding of peroxisomal motility. In addition, we propose some key future directions that may aid in the development of our understanding of peroxisomal trafficking.

## The machinery that elicits microtubule-dependent peroxisomal transport

### Early description of peroxisomal motility

A series of papers in the late 90s and early 2000s set out to characterise the motility of peroxisomes in mammalian cell lines using fluorescent markers targeted to the peroxisomal lumen [14, 16–19]. These early reports concluded similar characteristics of peroxisomal movement: namely, ~ 10% of peroxisomes undergoing fast directed trajectories with the other ~ 90% exhibiting slow, short-range displacements. Shorter-range peroxisomal motility is independent of the actin or microtubule cytoskeletons and it decreases at lower temperatures. Thus, this type of motion was dismissed as likely being diffusive behaviour of peroxisomes within the cytoplasm [19]. As such, shorter-range peroxisomal displacements have received little attention since these early studies. Conversely, long-range transport was found to depend on an intact microtubule cytoskeleton, as pharmacological disruption of microtubules completely abolishes these trafficking events [14, 16, 19]. Moreover, at any one time a large proportion of the peroxisomal population is aligned with microtubules [20–22]. Importantly, the characterisation of peroxisomal transport, distribution and microtubule alignment described by these early works has been confirmed in multiple different cell lines and held true as more advanced microscopy techniques have developed [23–26].

### How are peroxisomes linked to microtubules for long-range transport?

The exact mechanism of how peroxisomes couple to microtubules remains poorly characterised (see Table 1 for all known motors). Early work proposed a role for dynein through both in vitro microtubule binding assays and live imaging of peroxisomal trafficking in intact cells [14, 27]. Specifically, overexpression of dynamitin—a factor that is known to reduce dynein-dependent trafficking—or microinjection of an anti-dynein intermediate chain antibody significantly reduced long-range transport [14]. Overexpression of dynamitin also prevented the re-establishment of peroxisomal distribution following recovery from long-term disruption of microtubules by nocodazole treatment [14]. The most extensive characterisation of the motors required for peroxisomal transport is from work on microtubule-dependent motility in a *Drosophila* cell line [28]. Upon knockdown of several members of different kinesin families, including kinesin-1, kinesin-2, kinesin-3 and kinesin-14, only loss of kinesin-1 reduced peroxisomal trafficking [28]. This study also confirmed the role of dynein in peroxisomal transport as knockdown of dynein components abolished trafficking. The activity of kinesin-1 and dynein in peroxisomal dynamics has been shown to be interdependent in *Drosophila* [29]. It should also be noted that movement of microtubules themselves contributes to the net displacement of peroxisomes [20].

**Table 1:** Relationship of peroxisomes with motor proteins

Cytoskeleton	Motor	Model	References
Microtubules	Kinesin-1	*Drosophila* S2 cells	[28, 29]
	Dynein	Cos7 cells	[14]
		HepG2 cells	
		*Drosophila* S2 cells	[28, 29]
	KIFC3	Cos7 cells	[30]
		Adipocytes	[31]
		hTERT-RPE1 cells	[32]
	Adaptor		
	Pex14 (to dynein)	Human fibroblasts	[23]
	Pex1 (to KIFC3)	Cos7 cells	[30]
	Miro1	HeLa cells	[24]
		Cos7 cells	[26]
	TRAK2	HeLa cells	[24]
Actin	Myosin-II	CHO cells	[111]

Known motors and adaptors that have been implicated in the association of peroxisomes with both the microtubule and actin cytoskeletons in metazoans

The role for kinesin-1 in mammalian peroxisomal transport, however, is less clear. Knockdown of KIF5B (one of the three kinesin-1 family members) in mouse adipocytes has no effect on peroxisomal distribution. Instead, it has been shown that knockdown of the kinesin-14 family member, KIFC3, causes perinuclear clustering of peroxisomes in Cos7 cells and defects in fasting-induced changes in peroxisomal distribution in adipocytes [30, 31]. Moreover, knockout of KIFC3 perturbs peroxisomal motility and distribution in quiescent human retinal pigment epithelial cell line, leading to a reduction in cholesterol delivery to cilia [32]. Though this dramatic effect on distribution highlights a role for KIFC3 in peroxisomal positioning, whether peroxisomes use this motor for transport is not known. For example, knockdown of KIFC3 also causes a perinuclear distribution of mitochondria and ER [30]. Moreover, KIFC3 has been shown to have a role in the organisational dynamics of microtubules [33]. It would, therefore, be important to determine if KIFC3 directly transports peroxisomes, multiple organelles or the distribution changes are an indirect consequence of its role in microtubule dynamics. Furthermore, as KIFC3 is a minus-end mediated motor, resolving whether the plus-end mediated kinesin-1 family has a role in peroxisomal transport in mammals is required to fully understand how peroxisomal distribution is established.

**Fig. 1:**
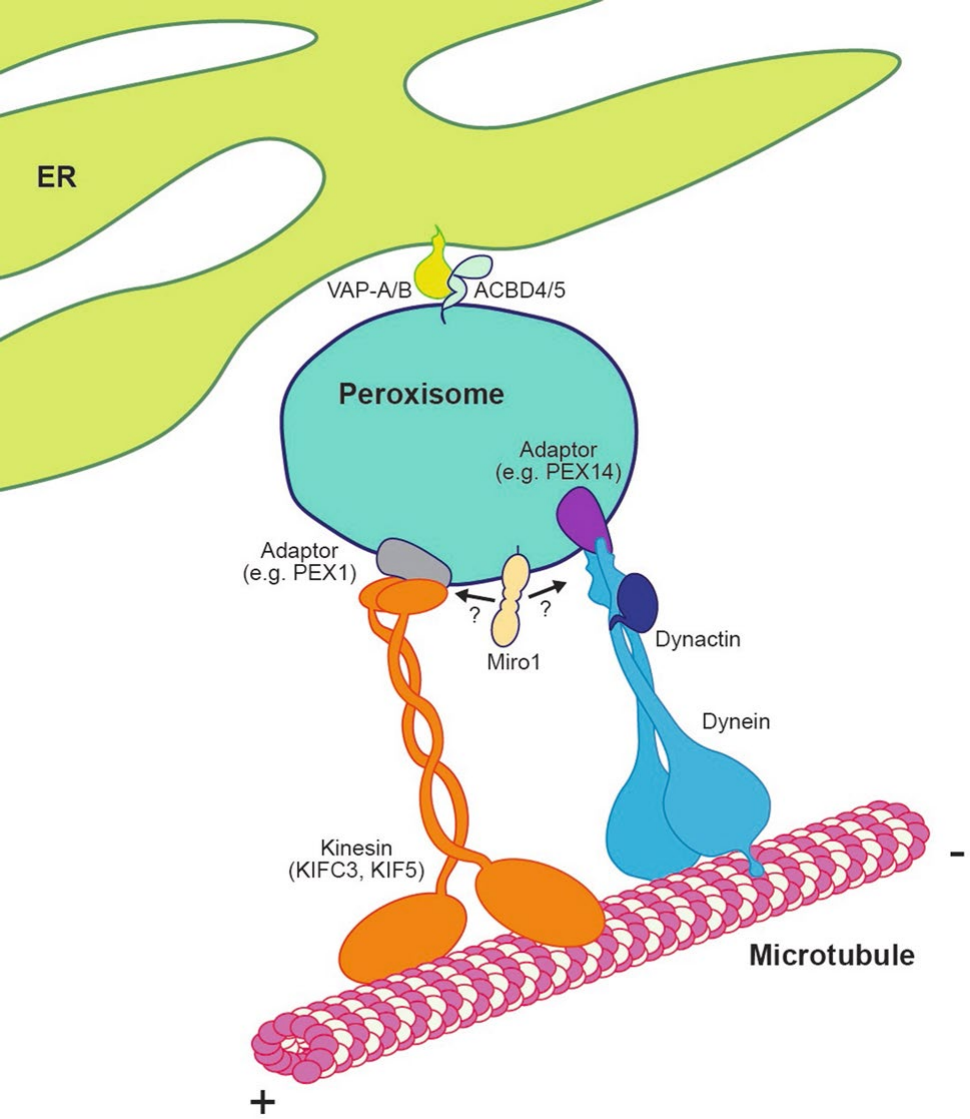
Peroxisomal trafficking machinery. Long-range trafficking is administered along the microtubules by the motor proteins dynein and kinesin (KIFC3, KIF5). The membrane protein PEX14, which is an essential part of the peroxisomal import machinery, allows the retrograde movement of peroxisomes along microtubules by interacting with dynein and its co-factor dynactin. Kinesins (KIFC3, KIF5) support the peroxisomal long-range trafficking towards the positive end of the microtubules by anchoring to the peroxisomal membrane using different adaptors (PEX1 for KIFC3). Miro1 is suggested to regulate peroxisomal trafficking by interacting with either the kinesin or dynein trafficking machinery. Oscillatory peroxisomal motility is dependent on tethering of the peroxisomes to the endoplasmic reticulum (ER) by the interaction of the ER membrane proteins VAP-A or VAP-B with the peroxisomal proteins ACBD4 or ACBD5

The promiscuity of the kinesin and dynein motors in intracellular trafficking requires specific membrane-anchored adaptors to recruit them from the cytoplasm to different organellar membranes. One candidate motor adaptor at peroxisomes is the protein Pex14 (Fig. 1). Pex14 is best characterised for its role in protein import into the peroxisomal lumen [34]. Interestingly, dynein, dynactin (a dynein co-factor) and β-tubulin have all been identified as Pex14 interactors through an unbiased mass spectrometry screen [23]. The importance of Pex14 in microtubule-dependent peroxisomal transport is illustrated by the loss of Pex14 leading to a reduction in long-range peroxisomal trafficking events, though the exact role of Pex14 in peroxisomal positioning is not yet fully defined [23, 25, 26]. For example, it has been proposed that Pex14 could be a microtubule docking factor for peroxisomes [35], as opposed to a motor adaptor. Furthermore, there is evidence that the N-terminus of Pex14—the proposed binding site for tubulin—resides in the peroxisomal lumen, i.e., may not have access to tubulin in the cytoplasm [36]. As a result, the exact role of the Pex14-dynein/dynactin interactions remains to be seen. Unlike loss of Pex14, patient-derived fibroblasts deficient in Pex1 or Pex5—two other peroxisomal protein import factors—show no difference in peroxisomal trafficking in comparison to control fibroblasts [23]. Loss of Pex1 or Pex13 has, however, been shown to cause a clustered peroxisomal distribution [22]. Moreover, Pex1 has been proposed to be the receptor for KIFC3 recruitment to peroxisomes [30]. These observations highlight the interplay of peroxisomal protein import with peroxisomal trafficking and distribution, though Pex14 is the only factor described as interacting with both the protein import and microtubule trafficking machineries. One interesting interpretation of the these data is that newly formed peroxisomes must be trafficked away following biogenesis to prevent peroxisomal clustering [22]. Indeed, intact microtubules are required for peroxisomal biogenesis [37].

To summarise, beyond the interactions of Pex14-dynein and Pex1-KIFC3, very little information exists on the mechanism of peroxisomal trafficking (see Table 1 for summary of all known motors and adaptors associated with peroxisomes and Fig. 1 for current model). More recently the Miro family of GTPases has also been proposed to modulate the coupling of peroxisomes to microtubules, similar to the previously reported roles for this protein at mitochondria [24, 26, 38].

## The impact of the Miro GTPases on peroxisomes

### Overlap of mitochondrial and peroxisomal homeostasis

**Fig. 2:**
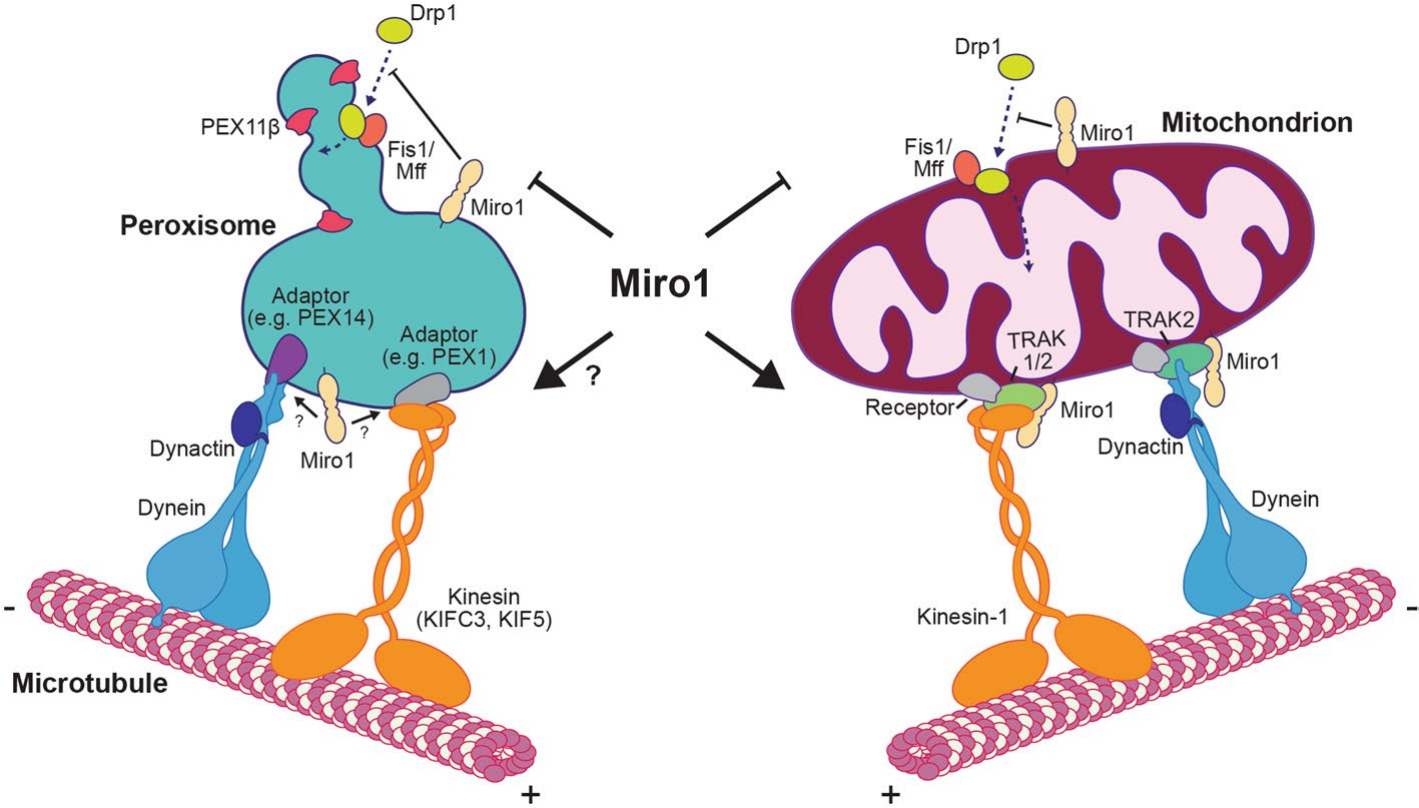
The role of Miro1 in peroxisomal and mitochondrial dynamics. Peroxisomes and mitochondria share a Drp1-dependent fission machinery, with the recruitment of Drp1 to either organelle being negatively regulated by Miro. Conversely, the microtubule-dependent transport of peroxisomes and mitochondria uses different machineries, thus it is currently unclear if the role of Miro1 on these processes is identical. Adaptors—unknown, likely PEX1 for KIFC3, TRAK2 for KIF5 and Pex14 for dynein; receptor—unknown, possibly mitofusins and Armcx1/3

The functions of peroxisomes have a significant overlap with mitochondria. Both organelles have roles in reactive oxygen species metabolism, lipid synthesis, β-oxidation of fatty acids and innate immunity requiring exchange of intermediates in a common pathway or, in some cases, expressing the same enzymes inside their matrices [39–42]. It has also been proposed that vesicles from mitochondria can target to peroxisomes and that mitochondria are important for peroxisomal biogenesis in mammalian cells [43, 44]. The interplay of these two metabolic organelles is further emphasised by the fact that they share part of their membrane proteome, including USP30, Mul1/MAPL, Fis1, Mff, GDAP1, Drp1, OMP25, Bcl-XL, Bcl-2, MAVS and Miro1/2 [8, 39, 44–50]. These proteins regulate many aspects of organelle homeostasis including, morphology, number and quality control. For example, both peroxisomes and mitochondria appear to use a similar machinery to carry out organellar fission (Fig. 2) [8, 45, 47, 50–52]. Fis1 and Mff are anchored in both the mitochondrial and peroxisomal membranes and recruit the GTPase Drp1 [45, 47, 53]. Once there, Drp1 causes constriction and ultimately scission of either organelle [52]. In the case of autophagic clearance, the deubiquitinase, USP30, has also been shown to negatively regulate the turnover of either organelle [48]. As the field of mitochondrial homeostasis is much more developed than that of peroxisomes, the shared proteome may allow one to draw analogy from mitochondrial to peroxisomal dynamics. As a result, the finding that Miro—a protein best characterised for its critical role in mitochondrial transport—is localised to peroxisomes was exciting for the field of peroxisomal motility [24, 26, 49]. Despite this observation, there has been conflicting data on the role of Miro at peroxisomes, which is discussed below.

### The role of Miro GTPases in mitochondrial transport

The Miro GTPases are a family of tail-anchored proteins, localised in the outer mitochondrial membrane with two calcium binding EF-hand domains and an N- and C-terminal GTPase domain residing in the cytoplasm [54, 55]. Work since the discovery of this family [54] has shown that Miro has a broad impact on mitochondrial homeostasis, distribution, morphology, ER-mitochondria contact sites, calcium buffering and mitophagy [56–67]. The best characterised of these roles is in regulating mitochondrial distribution through long range, microtubule-dependent mitochondrial transport [38, 68]. Like peroxisomes, mitochondria are trafficked using the molecular motors kinesin-1 and dynein (Fig. 2) [69–71]. The recruitment of these motors to the outer mitochondrial membrane is dependent on direct binding to the TRAK adaptors, with TRAK1 interacting with either kinesin or dynein and TRAK2 preferentially binding dynein [72–75]. Furthermore, TRAK1 may not simply recruit kinesin-1 to mitochondria, but also regulate its motor activity to promote motor processivity [76]. However, as neither TRAK adaptor has a transmembrane domain, it was assumed that the Miro proteins provide attachment of the trafficking machinery to the outer mitochondrial membrane. Recently, however, it has been shown that the TRAK proteins and both kinesin-1 and dynein can all localise to mitochondria in the complete absence of Miro [61]. Though Miro is not essential for the recruitment of the microtubule trafficking machinery, it still has a critical role in regulating mitochondrial trafficking. Loss of Miro leads to perinuclear clustering of mitochondria in the same manner as observed upon loss of TRAK, kinesin or dynein, in both mammalian and fly models [61, 69, 72, 73, 77–79]. It is, therefore, likely that the essential role of Miro in mitochondrial trafficking is to modulate transport events. For example, Miro facilitates the coordinated movement of inner and outer mitochondrial membrane by coupling the MICOS complex of inner mitochondrial membrane to the cytoskeleton [58]. In addition to long-range trafficking, Miro modulates short-range displacement on actin, by recruiting and stabilising the myosin motor, Myo19, to mitochondria [61, 80]. Hence, Miro proteins regulate mitochondrial position by coordinating microtubule- and actin-dependent motility. The characterisation of the mitochondrial trafficking machinery and its interplay with a variety of regulatory mechanisms, including calcium and glucose [81–84], make mitochondrial transport one of the best characterised organellar transport paradigms.

### Does Miro regulate peroxisomal motility and distribution?

The first descriptions of Miro on peroxisomes came from characterisation of tail-anchored proteins that localise to more than one of the following organelles in mammalian cells: ER, mitochondria and peroxisomes [49]. The localisation of Miro to peroxisomes has now been confirmed by multiple groups and found to be dependent on the cytosolic chaperone, Pex19 [24–26, 85]. It is possible that Miro can localise to peroxisomes in many species given that Gem1—the Miro orthologue in *Saccharomyces cerevisiae*—also localises to both mitochondria and peroxisomes [85]. Interestingly, there are several features within Miro that can modulate the extent of mitochondrial and peroxisomal localisation with either the loss of the first GTPase domain or the addition of exon 19 or 20 promoting peroxisomal localisation [24, 25]. Strikingly, inclusion of both exon 19 and 20 (Miro1 variant-4) leads to a near complete peroxisomal localisation of Miro1 [24, 25].

The well characterised role of Miro in maintaining mitochondrial distribution through long-range trafficking events, provides a hint as to its role at peroxisomes [61, 78, 81, 83, 86]. Indeed, two groups proposed a trafficking role for Miro1 at peroxisomes in quick succession [24, 26]. Both groups found that overexpression of Miro1 at peroxisomes (either through Miro1 variant-4 overexpression or Miro1 artificially localised to peroxisomes by the transmembrane domain of Pex26 and C-terminus of ALDP) led to clustering of peroxisomes, often at the periphery of the cell. Moreover, overexpression of peroxisomal Miro1 led to an increase in peroxisomal velocity and number of transport events. Okumoto et al*.* [24] also showed that knockdown of Miro1 leads to a decrease in peroxisomal trafficking. Additionally, overexpression of Miro1 variant-4 could recruit TRAK2 to peroxisomal membranes [24]. These observations, along with the early work showing that peroxisomal transport is dependent on kinesin-1 and dynein, like mitochondria, led to the conclusion that Miro1 likely shares a similar microtubule-dependent trafficking role at mitochondria and peroxisomes.

Surprisingly, despite the good evidence demonstrating that overexpression of Miro can alter peroxisomal distribution and microtubule-dependent peroxisomal motility, loss of Miro1 and Miro2—either solely or together in double knockout cells—leads to no detectable alteration in peroxisomal distribution [24–26, 87]. This finding is in complete contrast with mitochondria, which exhibit a dramatic relocalisation around the nucleus in the absence of Miro [61, 78, 87, 88]. Moreover, quantification of peroxisomal motility upon acute loss of Miro1 or chronic loss of Miro1, Miro2 or both Miro1 and Miro2 in mouse embryonic fibroblasts showed no change in long-range microtubule-dependent peroxisomal trafficking events in comparison to wild-type cells [25]. Instead, loss of Miro1 and Miro2 leads to a reduction in shorter-range peroxisomal motility, a type of trafficking that has been shown to be coupled to the endoplasmic reticulum [25, 89]. Importantly, loss of either KIF5B or TRAK1, proteins that control mitochondrial trafficking alongside Miro, also leads to no change in peroxisomal positioning or motility, despite leading to profound defects in mitochondrial distribution and transport [31, 70, 90]. It is, therefore, probable that the trafficking role of Miro at peroxisomes is unlikely to be completely analogous to that at mitochondria.

There are four other important considerations regarding any overlap in mitochondrial and peroxisomal transport. Firstly, Miro is near ubiquitous to eukaryotic life and is observed to have a diverse range of roles in mitochondrial homeostasis [56, 91, 92]. Consequently, Miro is not only a trafficking protein and, therefore, other roles for Miro at peroxisomes should be considered. One such role is in controlling peroxisomal number and morphology whereby Miro has been found to modulate peroxisomal size and number by negatively regulating Drp1-dependent fission [25]. Protein levels of Pex11β, a protein essential for peroxisomal elongation prior to fission, and Miro1 appear to be co-regulated [93]. Interestingly, it has been proposed that Miro1 may couple peroxisomes to microtubules to provide a driving force for peroxisomal elongation prior to fission [26]. This shape change could be achieved through a tug-of-war between directionally opposing motors on one organelle, as has been suggested for endosomes and mitochondria [94, 95]. It is also noteworthy that mitochondrial trafficking and fission/fusion dynamics have long been proposed to be linked [82, 96, 97] and that Drp1 recruitment to mitochondria is also modulated by Miro [25, 82]. As a result, there is good evidence for shared regulatory roles for Miro in mitochondrial and peroxisomal dynamics beyond establishing distribution through microtubule-dependent transport. Secondly, the architectures of mitochondrial and peroxisomal membranes are different. One role of Miro in mitochondrial trafficking is coupling the inner mitochondrial membrane to the cytoskeleton through its interaction with the MICOS complex [58]. By contrast, peroxisomes are single membrane structures and, thus, do not require membrane coupling. Thirdly, the distributions of peroxisomes and mitochondria are also strikingly different. For example, whereas mitochondria fill both the dendrites and axons of a neuron, peroxisomes are predominantly distributed in the somata and proximal dendrites [98]. Loss of Miro, TRAK1 or KIF5B also greatly impacts mitochondrial, but not peroxisomal, distribution [25, 31, 70, 90]. Finally, there are conditions where peroxisomal and mitochondrial motility do not react in the same way, such as high concentrations of reactive oxygen species halting microtubule-dependent mitochondrial, but not peroxisomal, transport [99, 100]. It would be interesting to see if calcium-dependent stopping of peroxisomes [19] is dependent on Miro, as in the case of mitochondria [81–83]. Considering this evidence, drawing strict analogy between mitochondrial and peroxisomal transport may be difficult, and therefore caution should be taken when assessing any shared machinery.

## Lessons from filamentous fungi

Filamentous fungi, such as *Aspergillus nidulans* and *Ustilago maydis* can serve as excellent models for studying organellar trafficking [101]. Like mammalian cells, they utilise microtubules for long-range transport using molecular motors like kinesin and dynein [102, 103]. Additionally, their long and thin cellular architecture is useful in tracking transport events. As microtubules are unidirectionally oriented at the hyphal tip (i.e., plus-end are polarised towards the periphery), kinesin- or dynein-dependent motion can also be delineated simply by the directional movement of organelles [102]. Work in these organisms found substantial similarities to mammalian cell lines, namely peroxisomes being homogenously distributed throughout the cell, with around 5% of peroxisomes undergoing long-range trajectories and the rest performing shorter-range motions [16, 19, 103, 104]. These long-range transport events are dependent on microtubules and deletion of either kinesin-1 or dynein perturbs peroxisomal distribution [102].

### Co-organellar trafficking of peroxisomes

Characterisation of microtubule-dependent trafficking in filamentous fungi also found that deletion of kinesin-3 causes peroxisomal accumulations [102]. Interestingly, upon probing the mechanism of kinesin-3 dependent peroxisomal motility, it was shown that these motions are actually peroxisomes “hitch-hiking” on early endosomes—a phenomenon that has been observed in two different species of fungi [103, 105]. The majority of peroxisomal movement on microtubules in fungi is driven by endosomal motility, which move using the adaptor hookA coupling to kinesin-3 [103, 106]. This endosome-dependent long-range peroxisomal motility also requires the presence of the linker protein PxdA [105]. Curiously, trafficking of lipid droplets and, to some extent, the ER is also dependent on the motility of early endosomes in filamentous fungi [103]. Thus, the co-organellar trafficking of peroxisomes may also be an important consideration in mammalian cells given the apparent role of the ER in peroxisomal motility. In mammals, most peroxisomes associate with the ER, with live imaging of the ER and peroxisomes showing that peroxisomes follow the oscillatory behaviour of the ER [25, 89]. Reduction in ER motility likely accounts for the decrease in peroxisomal movement observed upon loss of Miro [25]. Furthermore, loss of either VAP-A/B or ACBD4/5—proteins required for ER-peroxisome contact sites in mammalian cells—leads to an increase in peroxisomal motility, likely caused by the untethering of these organelles allowing freedom of peroxisomal movement [107–109]. As a result, a more deliberate look at the interdependence of peroxisomal motility with other organelles in mammalian cells may be informative.

### Role of actin cytoskeleton in maintaining peroxisomal distribution

Long-range directed trafficking on microtubules is a rapid way to alter the position of peroxisomes. However, it is known that alongside this movement, actin can play an important role in distributing organelles. Again, filamentous fungi have been useful in studying this phenomenon. In the complete absence of microtubules, peroxisomes localise at the hyphal tip in filamentous fungi [103, 106]. Interestingly, this phenomenon is dependent on the expression of the actin motor, myosin-5 [104]. It is important to note that peroxisomes were not found to be a myosin-5 cargo in filamentous fungi. Instead, it appears that active diffusion of cytoplasmic contents on F-actin and directed peroxisomal motility on microtubules may act in opposition to maintain a homogenous peroxisomal distribution. Evidence for the impact of opposing microtubule and actin driven peroxisomal motility was also seen in mammalian cells [104]. A similar phenomenon has also been observed in mammalian cells upon loss of Pex14. When Pex14-mediated microtubule-dependent peroxisomal trafficking is perturbed in patient fibroblasts, depolymerisation of actin drastically reduces the shorter-range oscillatory motion of peroxisomes [23]. This observation is in contrast to the robust data on actin perturbation, which shows no effect on peroxisomal motility under control conditions [16, 17, 19, 23, 25].

These results then raise the question, what is the full extent of the contribution of actin to peroxisomal distribution? In the case of plants and budding yeast, peroxisomal motility appears to be primarily driven by actin-myosin mediated motility, through class XI and class V myosins, respectively [110]. In mammals, myosin-II, RhoA and the actin-remodelling kinase ROCK have been proposed to localise at peroxisomes [111]. Additionally, peroxisomes have been shown to align with myosin-II filaments in these cells [111]. Therefore, it is possible that actin- and microtubule-dependent processes coordinate to maintain peroxisomal distribution.

Altogether, the work in filamentous fungi brings to light some important considerations when building a model of peroxisomal trafficking. It remains to be seen what the relative contribution of organelle contact sites, microtubules, actin and perhaps other cytoskeletons (e.g., septins and intermediate filaments) are, and how they differ between species and cell type.

## Factors that modulate peroxisomal transport and distribution

**Fig. 3:**
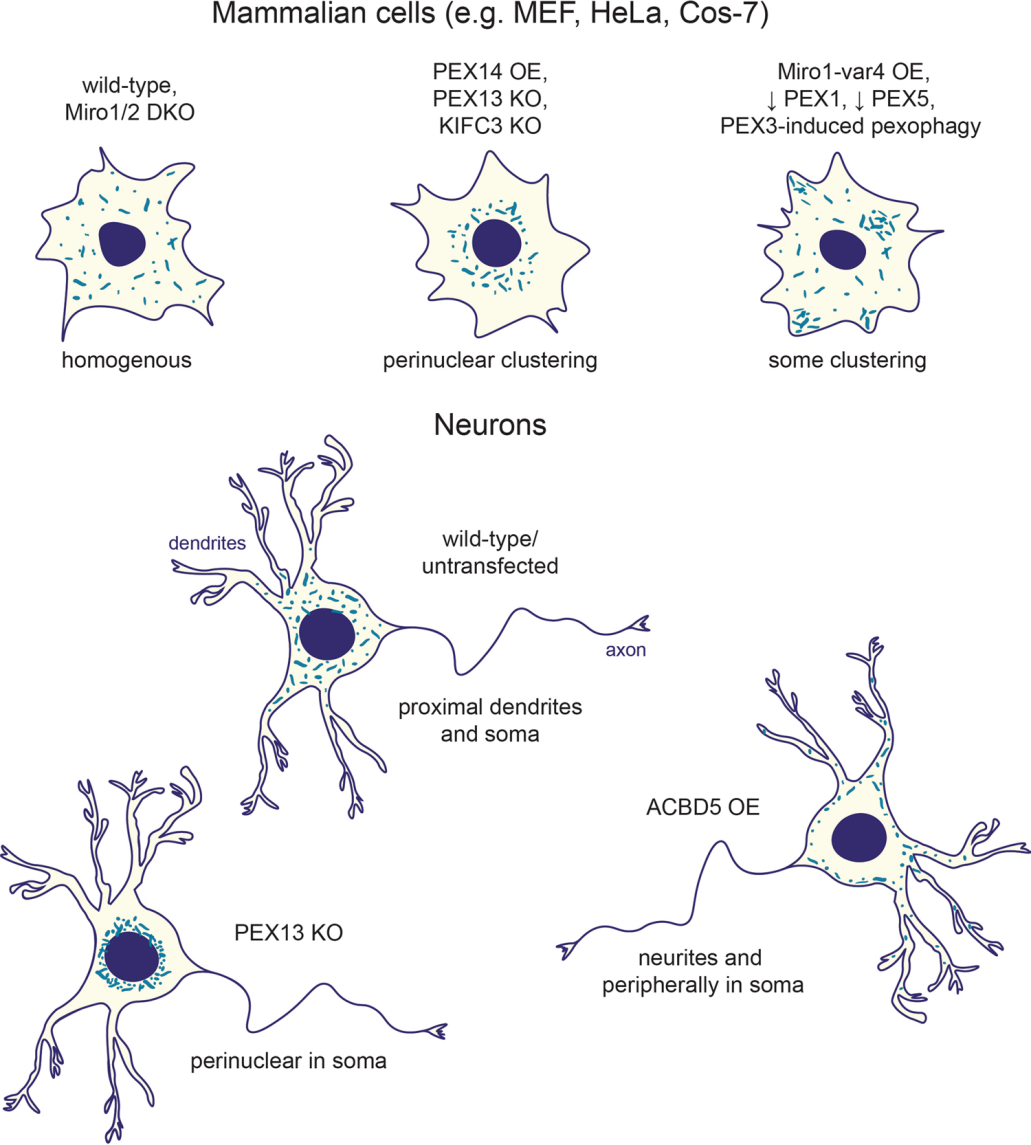
Factors affecting the distribution of peroxisomes in mammalian cells. Peroxisomes are distributed homogenously in several cell lines including Miro1/2 mouse embryonic fibroblasts. Depletion of members of the peroxisomal trafficking machinery (PEX14, KIFC3) causes a perinuclear positioning of peroxisomes, while changes in levels of other peroxisomal proteins lead to cytoplasmic clustering (e.g., overexpression of Miro1 variant 4, PEX1 or PEX5 deficiency, PEX3-induced pexophagy). In cultured neurons, peroxisomes are mainly found in the somata and proximal dendrites. Overexpression of ACBD5, however, leads to peroxisomal redistribution throughout the neurites and the periphery of somata. DKO, double knockout; OE, overexpression; KO, knockout; ↓, deficiency; var4, splice variant-4

The sections above outline our current understanding of the factors required to establish the distribution of peroxisomes (Fig. 3). For a real appreciation of the importance of peroxisomal trafficking to cellular health, however, we must understand the conditions which alter peroxisomal transport. In the case of mitochondria, high cytoplasmic calcium can halt motility, allowing mitochondria to buffer calcium and provide ATP for ion pumps [81–83, 112]. To date, several conditions have been identified that impact upon peroxisomal motility and positioning which may provide important insights into how the cell modulates peroxisomal distribution.

Neurons have proven instrumental in uncovering the machinery and regulatory mechanisms that control organelle position, as their extensive polar architecture means that organelles must often travel long distances to establish their distribution. They are, therefore, particularly susceptible to changes in organellar transport. Peroxisomes have been observed to predominantly localise to the somata and proximal dendrites of neurons and be largely absent from the axons (Fig. 3) [98, 113], although they likely undergo large changes to their localisation during development [114]. One potential regulatory mechanism for establishing peroxisomal distribution in neurons is through the proteins required for ER-peroxisome contact sites. More specifically, overexpression of ACBD5 in neurons causes a significant decrease in long-range peroxisomal transport events [98]. Furthermore, ACBD5 overexpression leads to peroxisomes distributing to the periphery of the somata and increasing their localisation throughout the neurites. The exact cause of this change in distribution is not known, though the authors propose it is likely separate from the ER-peroxisome contact role of ACBD5 [98].

Both ACBD5 and VAP-B are associated with pathologies, highlighting the importance of peroxisomal transport in human health (Table 2) [115, 116]. Another disease associated protein that has been studied in the context of peroxisomal transport is Spastin—a microtubule severing protein associated with hereditary spastic paraplegia. Peroxisomes have been shown to have reduced peroxisomal transport in *SPAST* mutant neuron-like cells [15, 117, 118]. The consequence of this defect in peroxisomal transport was a reduction in the number of peroxisomes in neurite processes and defects in handling cytoplasmic reactive oxygen species, leading to an increase in lipid peroxidation [15]. Interestingly, mutations in ABCD1 have also been shown to manifest in spasticity, like Spastin [119]. Spastin and ABCD1 have been shown to tether peroxisomes and lipid droplets together, to support fatty acid trafficking from lipid droplets to peroxisomes [120]. This is noteworthy as peroxisome-lipid droplet contact sites have recently been shown to be promoted by peroxisomal transport [31] and, therefore, Spastin may provide a means to control fatty acid metabolism through modulating peroxisomal trafficking and peroxisome-lipid droplet contact sites. Finally, overexpression of the Alzheimer’s disease associated protein, Tau, has also been shown to cause defects in peroxisomal distribution in neurites [121]. As a result, there is precedent for defects in peroxisomal transport forming part of the aetiology of diseases (Table 2).

**Table 2:** Peroxisomal transport in pathophysiology

Disease-associated protein	Model	Observed change	References
ACBD5 *Retinal dystrophy*	Knockdown in Cos7 cells	Increased oscillatory behaviour	[107]
	Knockdown in human fibroblasts	Increased oscillatory behaviour	[109]
	Overexpression in cultured neurons	Decreased long-range transport & Increased distribution through dendrites	[98]
VAP-B *Amyotrophic lateral sclerosis*	Knockdown in Cos7 cells	Increased oscillatory behaviour	[107]
Tau *Alzheimer’s disease*	Tau overexpression in differentiated N2a cells	Reduced peroxisomal abundance in neurites	[121]
Spastin *Hereditary spastic paraplegia*	*SPAST* mutant patient-derived, neuron-like cells	Reduced long-range transport & reduced peroxisomal distribution in neurites	[15]
Pex14 *Zellweger spectrum disorder*	Patient fibroblasts deficient in Pex14	Loss of long-range trafficking	[23]
Pex1 *Zellweger spectrum disorder*	Pex1 null patient fibroblasts	Peroxisomal clustering	[22]
Pex13 *Zellweger spectrum disorder*	Pex13 knockout mouse embryonic fibroblasts & Pex13 knockout cultured neurons	Perinuclear peroxisomal clustering	[22]

The impact of disease-associated proteins on peroxisomal trafficking and distribution in a wide range of model systems

There are a number of conditions that have been shown to impact peroxisomal motility. Firstly, the Just lab has probed the impact of a variety of pharmacological interventions on peroxisomal motility, identifying several that could reduce long-range peroxisomal transport. These include, increased cytosolic calcium, activation of phospholipase A_2_, co-stimulation of ATP and lysophosphatidic acid and inactivation of RhoA following treatment with exoenzyme C3 from *Clostridium botulinum* [17, 18, 111]. Peroxisomal distribution has also been shown to respond to changes in cellular physiology. For example, during fasting, peroxisomes traffic in KIFC3-dependent manner to form contacts with lipid droplets [31]. Blocking this trafficking-dependent association of peroxisomes and lipid droplets disrupts lipolysis, hinting that peroxisomal transport may be important in diseases with dysregulated lipid metabolism, e.g., obesity and type-2 diabetes. Additionally, peroxisomes have been shown to cluster during pexophagy, a process that is dependent on the ubiquitin receptor, p62 [122]. Interestingly, a similar role has been described for p62 in the autophagic clearance of mitochondria [123]. As p62 is essential for the clustering and functionality of late endosomal compartments, it is possible that either peroxisomal or mitochondrial clustering is required for efficient autophagolysosomal clearance of these organelles [124]. Though many conditions cause striking changes in peroxisomal motility and distribution, how these cellular signalling pathways feed into peroxisomal transport remains unknown. Moreover, it is not always clear if they are a direct or downstream consequence of the condition. Importantly, this means that it is difficult to know what the true extent of these, and other uncharacterised, alterations in peroxisomal distribution contribute to pathology.

## Summary and future directions

Work over the last 30 years has uncovered many aspects of how peroxisomal distribution is established through trafficking events. Here, we outline four main avenues that could build upon our current understanding towards a more comprehensive view of how peroxisomal functions are optimally positioned to support cellular health. Firstly, microtubule-dependent trafficking is required for establishing peroxisomal distribution. Crucially, however, how exactly the kinesin and dynein motors are recruited to the peroxisomal membrane is not defined. The best candidates to date are Pex14 and Miro1, though many details of how these two proteins control peroxisomal trafficking remain unknown. Whether the kinesin-1 family has a role in mammalian peroxisomal transport is also unclear and requires further research. Secondly, work in both filamentous fungi and mammalian cells has highlighted that organelle contact sites and the actin cytoskeleton can have a large influence on peroxisomal motility and positioning. Yet, what is the relative contribution of each of these mechanisms, alongside microtubules, in establishing peroxisomal distribution? Uncovering the way these players coordinate and interact with each other would help draw a clearer picture of the mechanisms of peroxisomal trafficking. In addition to the work aimed at uncovering the complexes that elicits peroxisomal transport, it will be essential to understand the conditions that modulate the movement. Few stimuli have currently been identified, and for those that have, it is not clear whether they have a direct or indirect effect. Knowing these conditions will inform further research and help the development of novel assays that explore peroxisomal trafficking. Finally, it may be important to focus future work into systems in which peroxisomes have particular importance in cellular function and, ultimately, organismal health. For example, oligodendrocytes, on account of their polarised morphology and essential function in producing the main lipid constituent of myelin, are one suitable model. Using cell types, which are more reliant on peroxisomal function, will make any perturbations have a more obvious effect on cellular biology and in turn aid the discovery of new mechanisms of regulation of peroxisomal trafficking. In summary, many characteristics of peroxisomal transport remain unsolved and will require attention if we are to fully understand the role that peroxisomal metabolic processes play in cellular physiology.
